# Juanbi Qianggu Formula inhibits fibroblast-like synovicytes activation via repressing LncRNA ITSN1-2 to promote RIP2 K48 ubiquitination

**DOI:** 10.1186/s13020-025-01164-4

**Published:** 2025-07-08

**Authors:** Yanqin Bian, Fang Li, Xinyu A, Zheng Xiang, Nanshan Ma, Jianye Wang, Boran Cao, Pengfei Xin, Xuan Cheng, Chang Liu, Bei Xiang, Jun Shen, Qigui Lu, Lianbo Xiao

**Affiliations:** 1 The Research Institute for Joint Diseases, Shanghai Academy of Traditional Chinese Medicine, Shanghai, 200052 China; 2 Guanghua Hospital Affiliated to Shanghai University of Traditional Chinese Medicine, Shanghai University of Traditional Chinese Medicine, Shanghai, 200052 China; 3 Shenzhen Hospital Affiliated to Shanghai University of Traditional Chinese Medicine, Shanghai University of Traditional Chinese Medicine, Shenzhen, 518001 China

**Keywords:** Rheumatoid Arthritis, Fibroblast, Like Synovicytes, Juanbi Qianggu Formula, lncRNA ITSN1, 2/miR-6823-3p/PELI3/RIP2 axis, Ubiquitination

## Abstract

**Background:**

Long non-coding RNA ITSN1-2(lncRNA ITSN1-2) promotes fibroblast-like synovicytes (FLS) proliferation and suppress apoptosis through activation of the NOD2/RIP2 signaling pathway, thereby exacerbating synovitis in Rheumatoid arthritis (RA) pathology. Juanbi Qianggu Formula (JBQG), a clinically efficacious traditional Chinese medicine, has shown significant efficiency in inhibiting FLS activation in RA and alleviating disease progression in RA patients. However, the molecular mechanism underlying JBQG's anti-arthritic effects remains incompletely understood, particularly regarding its potential to modulate lncRNA ITSN1-2-mediated NOD2/RIP2 signaling in FLS activation. This study aims to investigate the functional interplay between JBQG and the lncRNA ITSN1-2/NOD2/RIP2 axis in regulating FLS behavior during RA development.

**Methods:**

Synovial tissues were collected from 24 rheumatoid arthritis (RA) patients and 20 osteoarthritis (OA) patients to observe the lncRNA ITSN1-2/NOD2/RIP2 signal in RA synovial tissue and its correlation with RA inflammation and bone destruction. Blood-absorbed components of JBQG were analyzed through mass spectrometry, while network pharmacology and in vitro experiments were conducted to investigate JBQG's regulatory effects on NOD2/RIP2 signaling. Mechanistic studies focused on lncRNA-ITSN1-2/miR-2683-3p/PELI3/RIP2 interactions, employing dual-luciferase assays, FISH staining, and Co-IP/Western blot. To evaluate therapeutic efficacy, a collagen-induced arthritis (CIA) rat model with lncRNA ITSN1-2 overexpression was established. JBQG's effects were assessed through histopathological examination and serum inflammation factors analysis following 23 g/kg/day treatment for 4 weeks.

**Results:**

LncRNA ITSN1-2/NOD2/RIP2 signaling was significantly activated in RA synovial tissues, showing profound correlation with RA disease inflammation and progression. JBQG treatment reduced cytoplasmic lncRNA ITSN1-2 levels in FLS, thereby inhibiting NOD2/RIP2 pathway activation and FLS functions in migration and invasion. Mechanistically, lncRNA ITSN1-2 exerted competitive endogenous RNA (ceRNA) activity by sequestering miR-2683-3p, which upregulated PELI3 expression. This induction promoted RIP2 K48 ubiquitination, destabilizing RIP2 protein integrity and inhibiting downstream NF-κB signaling. Consequently, FLS migratory and invasive capacities were significantly diminished, underscoring JBQG's dual regulatory impact on lncRNA-miRNA cross-talk and inflammatory cytokine cascades.

**Conclusion:**

This study demonstrates that JBQG exerts potent anti-arthritic effects in RA therapy through dual regulatory mechanisms targeting the lncRNA ITSN1-2/miR-2683-3p/PELI3/RIP2 axis.

**Supplementary Information:**

The online version contains supplementary material available at 10.1186/s13020-025-01164-4.

## Introduction

Rheumatoid arthritis (RA) is a systemic autoimmune disorder predominantly manifesting as synovitis in small joints, with a prevalence of 0.37–0.42% in China [1], affecting approximately 5 million patients. Characterized by symmetrical joint swelling, pain, and morning stiffness in early stages, RA progresses to joint destruction, fusion, and deformity in advanced stages, ultimately leading to work disability. Despite multimodal therapy, RA remains chronic and incurable, with current treatments focusing on inflammation suppression and bone damage mitigation through biologics (e.g., TNF-α inhibitors) and disease-modifying antirheumatic drugs (DMARDs).

Traditional Chinese Medicine (TCM) has demonstrated prolonged clinical efficacy in RA management. Juanbi Qianggu Formula (JBQG), a herbal combination developed by National TCM Master Shi Qi, comprises five key ingredients: *Psoralea corylifolia* (15 g), *Drynaria fortunei* (15 g), *Epimedium brevicornu* (10 g), *Sinomenium acutum* (7.5 g), and *Angelica sinensis* (6 g). Through synergistic actions of kidney-bone strengthening, wind-dampness dispelling, blood circulation promotion, and analgesia, JBQG has shown significant clinical benefits in reducing inflammation (CRP, ESR) and improving Disease Activity Score in 28 joints using Erythrocyte (DAS28-ESR) in RA patients [2]. Preclinical studies further demonstrated JBQG’s anti-arthritic effects via FGFR1 signaling suppression, alleviating FLS invasion and bone erosion [3]. These findings underscore JBQG's therapeutic potential as a TCM-based complementary therapy for RA.

Long noncoding RNAs (lncRNAs) play pivotal regulatory roles in RA pathogenesis, modulating transcriptional and post-transcriptional processes [4, 5]. Our group identified lncRNA ITSN1-2, a novel RA-associated lncRNA, which is highly expressed in RA peripheral blood and correlates with inflammatory biomarkers (CRP, ESR, TNF-α, IL-17) and clinical scores (DAS28-CRP) [6, 7], Mechanistically, ITSN1-2 upregulation in RA synovium promotes NOD2/RIP2 pathway activation, inducing FLS proliferation and inhibiting apoptosis, thereby exacerbating synovitis [7].

This study elucidates a novel dual regulatory mechanism of JBQG. Upstream suppression: JBQG reduces cytoplasmic lncRNA ITSN1-2 levels in FLS, derepressing miR-2683-3p to upregulate PELI3 expression. Downstream blockade: PELI3 upregulation promotes RIP2 K48 ubiquitination, destabilizing RIP2 protein and inhibiting NF-κB nuclear translocation (phosphorylated IKB/p65 reduction). This two-pronged strategy not only alleviates synovitis and bone destruction but also highlights JBQG's precision medicine potential for RA patients with high ITSN1-2 expression. Furthermore, it provides a theoretical foundation for TCM-biologic combination therapies (e.g., JBQG + NOD2 inhibitors), addressing current limitations of single-target treatments. Future studies should prioritize long-term safety assessments, clinical biomarker validation, and mechanistic studies to advance RA therapeutics.

## Materials and methods

### Human synovial tissues

Synovial tissues were prospectively collected from 24 RA patients and 20 OA patients undergoing knee arthroplasty, with demographic matching for gender and age (p < 0.05). Detailed clinical characteristics are systematically documented in Table 1. Preoperative evaluations included DAS28-ESR score, CRP and ESR levels, and knee joint radiography to assess disease severity and joint structural damage. Synovial specimens were carefully excised to remove adherent adipose tissue using microsurgical techniques. Tissues were partitioned into cryopreservation aliquots (30–50 mg per tube) in RNase-free tubes. Then samples were immediately immersed in liquid nitrogen for 10 s to achieve ultra-rapid freezing, followed by long-term storage at − 80 °C until downstream analysis. The study was conducted under approval from the Ethics Committee of Guanghua Hospital Affiliated to Shanghai University of Traditional Chinese Medicine (Approval No.: 2018-K-12), with informed consent obtained from all participants prior to enrollment.

**Table 1:** Clinical Characteristics of the Enrolled Participants

	RA	OA	p value
Gender (F/M)	20/4	16/4	> 0.9999
Age (year, Mean + SD)	71.79 + 7.82	76.61 + 7.87	0.07
CRP (mg/L, Mean + SD)	34.32 + 40.54	7.85 + 12.50	*0.0076*
ESR (mm/h, Mean + SD)	61.63 + 33.03	23.7 + 22.81	< *0.0001*
TJC (n, Meas + SD)	2.46 + 2.0	–	–
SJC (n, Mean + SD)	1.54 + 1.98	–	–
PGA (mm, Mean + SD)	41.86 + 22.59	–	–
DAS28-ESR (Mean + SD)	4.50 + 0.56	–	–

TJC, Tender Joint Count; SJC, Swollen Joint Count; DAS28-ESR, PGA, Patient Global Assessment; Disease Activity Score-28 Joints

DAS28-ESR = (0.56*SQRT(TJC)) + (0.28*SQRT(SJC)) + (0.7*LN(ESR)) + (0.014*(PGA))

### Knee joint modified sharp/van der Heijde score (mSS)

Knee joint bone damage was assessed based on X-rays of the enrolled patients, using a scoring system modified from Reference [8]. The scoring method consisted of two components: bone erosion score (ES) and joint space narrowing (JSN) score. The medial and lateral femoral condyles (distal femur) and the medial and lateral tibial plateaus (proximal tibia) were evaluated for ES scores. Each region was scored on a scale of 0–5 as follows: 0 points: No bone erosion; 1 point: Mild erosion, with minimal cortical damage; 2 points: Erosion affecting up to 50% of the cortical thickness; 3 points: Erosion exceeding 50% of cortical thickness, but the bone structure remains intact; 4 points: Severe erosion, with significant destruction approaching full thickness; 5 points: Complete bone erosion or structural collapse. A maximum score of 5 points per region was assigned, resulting in a total maximum ES of 20 points for all four regions. The medial compartment (medial femoral condyle and medial tibial plateau) and the lateral compartment (lateral femoral condyle and lateral tibial plateau) were evaluated for JSN scores. Each compartment was scored on a scale of 0–4 as follows: 0 points: Normal joint space; 1 point: Mild narrowing; the joint space is reduced but still visible; 2 points: Moderate narrowing, with significant reduction in joint space; 3 points: Severe narrowing, with the joint space nearly obliterated; 4 points: Complete loss of joint space with evidence of bony ankylosis. A maximum of 4 points per compartment was assigned, resulting in a total maximum JSN of 8 points for both compartments. The total mSS was calculated as the sum of the ES and JSN scores. Radiographic evaluations were independently conducted by two radiology professionals. The final score for each component was determined by taking the average of the two evaluators' scores, ensuring consistency and reliability.

### Analysis of blood-absorbed components of JBQG

**Fig. 1:**
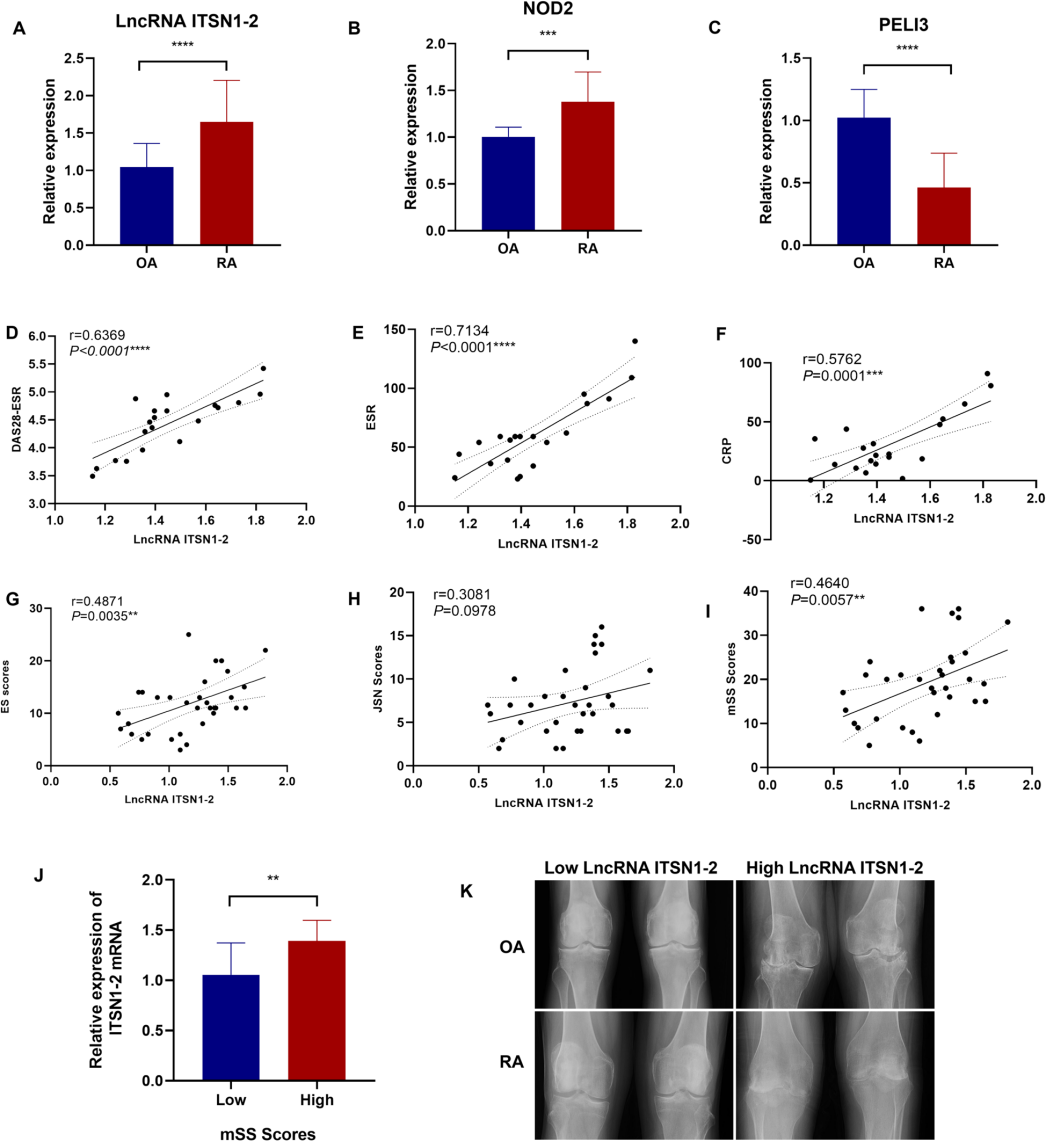
Relationship Between LncRNA ITSN1-2 and Joint Inflammation and Bone Destruction. **A–C** Relative expression levels of lncRNA ITSN1-2, NOD2, and PELI3 mRNA in synovial tissues of RA patients (n = 24) and OA patients (n = 20); **D–F** Correlation between lncRNA ITSN1-2 mRNA expression levels and DAS28-ESR, ESR, and CRP in RA patients (n = 20); **G–I** Association of lncRNA ITSN1-2 with knee joint bone damage ES scores, JSN scores, and total mSS scores in patients (n = 44); **J** Comparison of lncRNA ITSN1-2 levels between patients with low and high mSS scores; **K** Joint X-ray comparison between OA and RA patients with low and high levels of lncRNA ITSN1-2. T-tests were used for comparisons between two groups, and pearson correlation analysis was used for correlation studies. **p < 0.01; ***p < 0.001; **p < 0.0001 compared with the indicated group

Twelve SD rats (210 ± 10 g), female, were randomly assigned to experimental and control groups. All rats were fasted for 12 h with free access to water. The control group (n = 3) received 0.4% CMC-Na solution via gavage, while the experimental group was administered JBQG solution at a dose of 21.40 g crude drug/kg, prepared as 1.07 g crude drug/mL in 0.4% CMC-Na-equivalent to four times the human dose for a 60 kg adult. The quality control of JBQG solution follows our previous report [2]. The testing follows the methods specified in the “Chinese Pharmacopoeia 2020”, with analytical results confirming pharmacopeial compliance for all constituents: *Psoralea corylifolia*: the total content of Psoralen and isopsoralen is 0.70%; *Drynaria fortunei*: the content of Hesperidin is 0.86%; *Angelica sinensis*: the content of Ferulic acid is 0.072%; *Epimedium brevicornu*: the total content of Icariin, icaritin, and desmethylicaritin, and epimedin is 1.5%; *Sinomenium acutum*: the content of corynoline is 1.09%. Supple. Figure 1 presents the complete quality analysis data.

Blood samples were collected from the abdominal aorta under 3% pentobarbital anesthesia at 0.5, 1, and 1.5 h post-administration (n = 3 per time point) into non-anticoagulant tubes. The samples were centrifuged at 6000*g*, 4 °C for 15 min to obtain serum. A 100 μL serum aliquot was mixed with 500 μL methanol to precipitate proteins, followed by centrifugation at 10,000*g*, 4 °C for 15 min. Subsequently, 480 μL of the supernatant was transferred to a 1.5 mL tube, dried under a nitrogen stream at 37 °C, and reconstituted with 80 μL of 20% methanol aqueous solution. After a final centrifugation at 10,000*g* for 15 min, the supernatant was collected for analysis. Serum samples before and after administration were analyzed using UHPLC-Q-Orbitrap HRMS, as shown in Supplementary Fig. 2 and 3. A total of 44 blood-absorbed components were identified, with detailed results presented in Table. 2.

**Table 2:** Identification of 44 Active Blood-Absorbed Components

No	Structural Formula	Name	Sources	Types	0.5 h (peak area/10^5^)	1 h (peak area/10^5^)	1.5 h (peak area/10^5^)
1	C_7_H_12_O_6_	Quinic acid	DG	Organic Acids	308.7	215.2	398.3
2	C15H14O6	Epicatechin	GSB/BGZ	Flavonoids	39.8	46.4	37.5
3	C_18_H_19_NO_3_	Tsuduranine	QFT	Alkaloids	9.0	5.9	11.8
4	C_18_H_21_NO_3_	*N*-methylcoclaurine	QFT	Alkaloids	2.8	2.5	3.0
5	C_19_H_25_NO_4_	Dihydrosinomenine	QFT	Alkaloids	233.3	183.9	29.0
6	C_18_H_21_NO_5_	Amberline	QFT	Alkaloids	8.8	5.2	13.5
7	C_18_H_21_NO_4_	*N*-demethylsinomenine	QFT	Alkaloids	1619.0	1740.0	1891.0
8	C_19_H_23_NO_4_	Sinomenine	QFT	Alkaloids	5748.4	3906.2	6552.9
9	C_38_H_46_N_2_O_8_	7^′^8^'^-dihydro-1,1^'^-disinomenine	QFT	Alkaloids	41.2	18.3	47.5
10	C_24_H_31_NO_9_	3'-hydroxy-*N*-methyl coclaurine-1-*O*-glucoside	QFT	Alkaloids	3.4	2.9	1.7
11	C_19_H_21_NO_4_	Discretamine	QFT	Alkaloids	60.2	42.0	92.2
12	C_23_H_29_NO_8_	Coclaurine glucoside	QFT	Alkaloids	3.7	3.8	4.3
13	C_19_H_24_ClNO_6_	Acutumine	QFT	Alkaloids	52.5	24.9	42.0
14	C_20_H_23_NO_4_	Magnoflorine	QFT	Alkaloids	566.8	502.4	583.7
15	C_18_H_19_NO_3_	Stepharine	QFT	Alkaloids	50.6	29.4	79.5
16	C16H18O8	3-O-p-coumaroylquinic acid	YYH/DG	Flavonoids	3.7	3.3	4.0
17	C_12_H_16_O_5_	Senkyunolide S	DG	Phthalides	113.6	97.1	77.8
18	C17H18O9	Psoralenoside	BGZ	Coumarins	455.3	590.4	983.8
19	C17H18O9	Isopsoralenoside	BGZ	Coumarins	673.0	593.6	1050.5
20	C_12_H_16_O_5_	Senkyunolide R	DG	Phthalides	2.8	3.0	4.0
21	C_7_H_6_O_3_	Salicylic Acid	DG	Organic Acids	1356.5	2313.4	1436.2
22	C_20_H_23_NO_5_	Isocorydine *N*-oxide	QFT	Alkaloids	0.6	0.4	1.0
23	C_19_H_21_NO_5_	*N*-trans-feruloylmethoxytyramine	QFT	Alkaloids	7.6	5.4	9.8
24	C_12_H_16_O_3_	Senkyunolide G	DG	Phthalides	156.1	88.7	96.3
25	C_12_H_14_O_3_	4-Hydroxy-3-Butylphthalide	DG	Phthalides	367.1	142.3	251.2
26	C_11_H_6_O_3_	Psoralen	BGZ	Coumarins	62,279.7	18,848.9	54,208.0
27	C_19_H_19_NO_4_	Cheilanthifoline	QFT	Alkaloids	19.4	16.5	23.4
28	C_18_H_19_NO_4_	*N*-feruloyltyramine	QFT	Alkaloids	28.7	4.2	11.6
29	C_11_H_6_O_3_	Angelicin	BGZ	Coumarins	24,972.0	10,556.5	25,339.5
30	C20H20O6	Brosimacutin G	BGZ	Flavonoids	12.9	6.3	8.6
31	C_24_H_34_O_9_	[4-(β-d-glucopyranosyloxy)-2,6-bis(3-methyl-2-buten-1-yl)phenyl](hydroxy)acetic acid	YYH	Flavonoids	3.7	2.8	3.4
32	C_12_H_12_O_2_	(Z)-Butylidenephthalide	DG	Phthalides	2298.2	765.5	1208.1
33	C_12_H_14_O_2_	3-Butylphthalide	DG	Phthalides	185.0	65.3	100.1
34	C21H22O5	Ashitaba-chlcone	BGZ	Flavonoids	21.9	16.0	17.7
35	C_34_H_42_O_17_	Icariin	YYH	Flavonoids	3.3	1.8	0.9
36	C20H16O5	Psoralidin	BGZ	Flavonoids	9.9	4.7	5.7
37	C18H22O	Bakuchiol	BGZ	Other	54.5	32.6	76.0
38	C20H20O4	isobavachalcone	BGZ	Flavonoids	4.2	/	1.5
39	C_18_H_13_NO_4_	6-*O*-demethylmenisporphine	QFT	Alkaloids	463.9	347.1	739.0
40	C20H18O4	Neobavaisoflavone	BGZ	Flavonoids	23.4	9.3	13.0
41	C20H18O4	4-hydroxyisolonchocarpin	BGZ	Flavonoids	1.6	1.0	0.6
42	C20H18O4	Isobavachromene	BGZ	Flavonoids	5.0	2.0	1.6
43	C_27_H_30_O_10_	Baohuoside I	YYH	Flavonoids	10.8	8.8	7.2
44	C_19_H_15_NO_5_	Dauriporphinoline	QFT	Alkaloids	6.8	9.2	4.0

### Cell culture

The primary cell isolation procedure was performed following the method described in the literature [9]. Briefly, synovial tissue was collected, minced into small pieces, and transferred into DMEM solution containing 3 mg/mL collagenase. The tissue was digested in a shaker incubator at 37 °C for 2 h. The isolated cells were then centrifuged and resuspended in complete DMEM medium supplemented with 10% FBS and 1% penicillin–streptomycin. The cells were cultured in a 37 °C incubator with 5% CO₂, with the culture medium replaced every 2–3 days. Cells at passages 3–5 were used for subsequent experiments. MH7A cells were stimulated with 10 ng/mL TNF-α and 10 ng/mL IL-17 for 24 h, or with 100 ng/mL TNF-α for 24 h to achieve activation. For lncRNA overexpression or knockdown experiments, lncRNA overexpression or knockdown vectors were introduced into the cells 48 h prior to TNF-α stimulation. Following TNF-α stimulation for 24 h, JBQG intervention was applied by treating the cells with 10% medicated serum, 10% control serum, miRNA mimics, or lncRNA-related treatments.

### Cell migration and invasion assay

Cell migration and invasion assays were performed using transwell chambers with an 8 μm pore size. Migration assays conducted without matrigel, while invasion assays required matrigel coating of the upper chamber. Cells were digested and prepared as a suspension at a concentration of 5 × 105 cells/mL. For the migration assay, 200 μL of serum-free cell suspension was added to the upper chamber, and 600 μL of DMEM medium containing 10% FBS was added to the lower chamber to serve as a chemoattractant. In the invasion assay, 50 μL of matrigel was applied to the upper chamber and incubated at 37 °C for 1 h to solidify before seeding the cells in the same manner. The cells were incubated at 37 °C with 5% CO₂ for 24–48 h. After incubation, non-migrated or non-invaded cells were gently removed from the upper chamber using a cotton swab, and the membrane was washed twice with PBS. The cells were then fixed with 4% paraformaldehyde for 15 min. Following fixation, the cells were stained with crystal violet for 15 min, washed with PBS to remove excess stain, and air-dried. Finally, migrated or invaded cells were observed and counted under a microscope. Experimental data were analyzed using ImageJ software, and statistical analyses were performed to evaluate cell migration and invasion capabilities.

### Cell counting kit-8 (CCK-8) assay

The CCK-8 assay was conducted to assess cell viability. Cells were seeded into a 96-well plate at a density of 2 × 10^3^ cells per well and incubated at 37 °C with 5% CO₂ until adherence. After intervention with 1%, 5%, and 10% medicated serum for 24, 48, 72, and 96 h, the supernatant was removed, and 90 μL of fresh DMEM along with 10 μL of CCK-8 reagent were added to each well, ensuring thorough mixing without generating bubbles. The plate was then incubated at 37 °C with 5% CO₂ for 2 h. Absorbance at 450 nm was measured using a microplate reader, and the resulting optical density (OD) values were used to evaluate cell viability. Data analysis was performed to generate growth curves and conduct statistical evaluations.

### ELISA

The expression levels of MMP-1 and MMP-3 in cell culture supernatants, as well as TNF-α, IL-6, and IL-17 in rat serum, were measured using ELISA kits. The assay was conducted according to the manufacturer’s instructions. After completing the ELISA procedure, absorbance values were recorded using a microplate reader, and the data were analyzed to determine the concentration of each target protein.

### Real-time PCR

The relative expression levels of lncRNA, miRNA, and NOD2/RIP2 pathway-related genes in activated MH7A cells were measured using real-time PCR. Total RNA was extracted using the Trizol method and quantified with a NanoDrop 2000 spectrophotometer (Thermo Scientific). Reverse transcription was performed using the HiScript® II Q RT SuperMix kit (VAZYME) for mRNA and the HiScript II Q Select RT SuperMix kit (VAZYME) for miRNA, both of which include a gDNA wiper. PCR amplification was conducted using the ChamQ SYBR qPCR Master Mix kit (VAZYME). β-actin was used as the internal control. The primer sequences used for amplification are showed Table 3. The relative expression levels of target genes were calculated according to the 2^−ΔΔCt^ formula.

**Table 3:** Primer Sequences List

Gene	Primer	Sequence (5'–3')
Homo b-actin	Forward	GCACTCTTCCAGCCTTCCTT
	Reverse	TTCATTGTGCTGGGTGCCA
Homo LINC00649	Forward	AGCCCCATTTTGGAGGCAC
	Reverse	GGGCGTCTTCAGGTTGTCTC
Homo PELI3	Forward	TCTTGCGTTCTCTCCTCTCCC
	Reverse	CTCGGTTTCCTCACCTCCTTC
Homo NOD2	Forward	TCCGCAAGCACTTCCACTCCA
	Reverse	CTCCACGCCAATGTCACCCAC
Homo RIP2	Forward	CGCTCACGTTCTTATTCTTGC
	Reverse	TCTGTTTTCTCTTGGTGGCTC
U6	Forward	CGCTTCGGCAGCACATATAC
	Reverse	AAATATGGAACGCTTCACGA
Hom MIR223	Loop primer	GTCGTATCCAGTGCAGGGTCCGAGGTATTCGCACTGGATACGACTGGGGTAT
	F primer	TGCGCTGTCAGTTTGTCAAATA
Homo MIR532	Loop primer	GTCGTATCCAGTGCAGGGTCCGAGGTATTCGCACTGGATACGACTGCAAGCC
	F primer	TGCGCCCTCCCACACCCAAGGC
Homo MIR6823	Loop primer	GTCGTATCCAGTGCAGGGTCCGAGGTATTCGCACTGGATACGACTGGAGGGA
	F primer	TGCGCTGAGCCTCTCCTTCC

### Dual-luciferase reporter assay

We conducted a dual-luciferase reporter assay to explore the binding interactions between lncRNA ITSN1-2 and miRNA-6823-3p, as well as PELI3 and miRNA-6823-3p. Cells were seeded into 12-well plates and co-transfected with luciferase reporter plasmids containing either the wild-type or mutated binding sites of ITSN1-2 and PELI3, along with the miRNA-6823-3p mimic and NC mimic as an internal control, using Lipofectamine™ 2000 (Invitrogen). After 48 h of transfection, cells were lysed with 300 μL of passive lysis buffer. According to the manufacturer's instructions, 100 μL of the lysate was transferred to a luminometer plate, followed by the sequential addition of 100 μL of firefly luciferase assay reagent to measure firefly luciferase activity and 100 μL of Renilla luciferase assay reagent to measure Renilla luciferase activity. The relative luciferase activity was determined by normalizing firefly luciferase to Renilla luciferase, and the results were analyzed to assess the binding interactions between ITSN1-2, PELI3, and miRNA-6823-3p.

### Fluorescence in situ hybridization assay

We performed a fluorescence in situ hybridization (FISH) assay to observe the localization of lncRNA ITSN1-2 and miR-6823-3p in FLS cells, as well as their co-localization sites. Cells were seeded into 6-well plates and stimulated with 100 ng/mL TNF-α for 24 h. Following stimulation, the cells were treated with 10% medicated serum or control serum for an additional 24 h. After treatment, the supernatant was removed, and the cells were washed with PBS, followed by fixation with 4% paraformaldehyde at room temperature for 15 min. The fixed cells were washed and permeabilized at 4 °C for 15 min, then dehydrated using a graded ethanol series. The cells were incubated overnight at 37 °C in hybridization buffer containing the fluorescent probes, protected from light. The next day, unbound probes were removed by washing with SSC buffer, and the nuclei were counterstained with DAPI. Finally, the coverslips were mounted with antifade mounting medium, and fluorescence signals were visualized under a fluorescence microscope (Leica Microsystems). The results were analyzed to determine the subcellular localization and interactions of the target RNA.

### Co-immunoprecipitation

Cells were washed twice with pre-chilled PBS and lysed with IP lysis buffer containing protease and phosphatase inhibitors (Meilunbio, MB12707) on ice for 15 min. After centrifugation at 10,000 rpm for 10 min at 4 °C, the supernatant was collected, and protein concentration was measured. A total of 500 µg protein was adjusted to 500 µL with PBS. Agarose Protein A + G beads (Beyotime, P2055) were washed, prepared to a 50% concentration, and used to remove non-specific binding by incubating with the sample at 4 °C for 2 h. After removing the beads, 4 µg of RIP2 antibody (Proteintech, 15,366–1-ap) was added and incubated overnight at 4 °C. The next day, fresh beads were added for 6 h at 4 °C to pull down protein complexes. The beads were washed with PBS, resuspended in loading buffer, boiled for 5 min, and centrifuged. The supernatant and was analyzed by SDS-PAGE and Western blot to detect proteins of K48Ub (PTMBIO, TM-7205), K63Ub (PTMBIO, TM-7228), P65 (Proteintech, 10,745–1-AP), P-IKB (Affinity, AF2002), MMP3 (Affinity, AF0217) and β-actin (Proteintech, 66,009–1-Ig).

### Animal experiment

Six-week-old female SPF SD rats (180 ± 20 g) were randomly assigned to six groups (n = 8 per group): AAV(−), AAV( +), AAV(−) + CIA, AAV( +) + CIA, AAV(−) + CIA + JBQG, and AAV( +) + CIA + JBQG. The CIA model was established based on the method described by Meng et al. [10]. All rats in the CIA groups received an initial subcutaneous injection of 0.3 mL of an emulsion containing bovine type II collagen and complete Freund’s adjuvant at the base of the tail. A booster injection of 0.2 mL of the same emulsion was administered at the same site on the opposite side seven days later. Control group rats were injected with an equal volume of normal saline as a sham immunization. On the day of the initial immunization, all AAV( +) rats received intra-articular injections of ITSN1-2 overexpression adenovirus (10◊^12^ vg/mL, 20 μL per joint) in both knee joints, while AAV(−) rats were injected with an equivalent volume of control virus. Two weeks after the booster immunization, JBQG group rats received JBQG solution via gavage at a dose of 23 g/kg/day, administered once daily for four consecutive weeks. The remaining groups were gavaged with an equivalent volume of distilled water. Throughout the experiment, body weight, ankle joint diameter, and paw thickness were measured weekly to monitor joint swelling. After four weeks of treatment, the rats were euthanized for sample collection. Peripheral blood serum was collected for inflammatory cytokine analysis, and knee joint samples were harvested for histological examination using HE and Safranin O staining to assess local inflammation and cartilage changes.

### Statistical analysis

In this study, statistical analyses and data visualization were performed using GraphPad Prism 8 software. Student’s t-test was used for comparisons between two groups, while one-way ANOVA and two-way ANOVA, followed by Tukey’s post hoc test and Tukey’s multiple comparisons, were employed for comparisons among three or more groups. Pearson correlation analysis was conducted to assess relationships between variables. Data were expressed as mean ± standard deviation (mean ± SD), and a p-value of less than 0.05 (p < 0.05) was considered statistically significant.

## Results

### LncRNA ITSN1-2 modulates NOD2/RIP2 signaling to promote synovial inflammation and joint damage in rheumatoid arthritis

Previous studies have demonstrated that lncRNA ITSN1-2 play a critical role in inflammatory responses, disease severity, and poor prognosis across multiple disorders [11–13]. Our prior work demonstrated that lncRNA ITSN1-2 enhances NOD2/RIP2 signaling, promoting FLS proliferation and inhibiting apoptosis, thereby exacerbating synovial inflammation in RA [7]. To further elucidate its role, we analyzed ITSN1-2, NOD2, and PELI3(an enzyme can regulate RIP2 ubiquitination) expression in synovial tissues from 24 RA patients and 20 OA patients (demographically matched for age and gender). As shown in Fig. 1A–C, ITSN1-2 and NOD2 expression were significantly upregulated where PELI3 was downregulated in synovial tissues compared to OA. Then we observed the relationship between lncRNA ITSN1-2 and inflammation. As shown in Fig. 1D–F, ITSN1-2 levels positively correlated with RA disease activity markers, including DAS28-ESR, ESR, and CRP. These findings reinforce our earlier hypothesis that lncRNA ITNS1-2 drives NOD2/RIP2-mediated inflammation in RA [6]. Notably, the association between elevated lncRNA ITSN1-2 expression and increased joint damage-evidenced by higher erosion score (ES) and total Modified Scoring system (mSS) scores-was observed in both RA and OA patients (Fig. 1G–K). This cross-disease correlation suggests that lncRNA ITSN1-2 may act as a common mediator in inflammatory-driven joint degeneration, transcending autoimmune etiologies. Such findings position ITSN1-2 as a promising noninvasive biomarker for assessing synovial inflammation and structural damage severity in inflammatory arthritis. Moreover, its differential regulation in RA synovium (upregulation) versus OA (less pronounceds) highlights its specific pro-inflammatory role in autoimmune disease, warranting further investigation into its utility in monitoring disease progression and therapeutic response.

### JBQG modulates the lncRNA ITSN1-2/PELI3 signaling axis to inhibit NOD2/RIP2 pathway activation in RA.

**Fig. 2:**
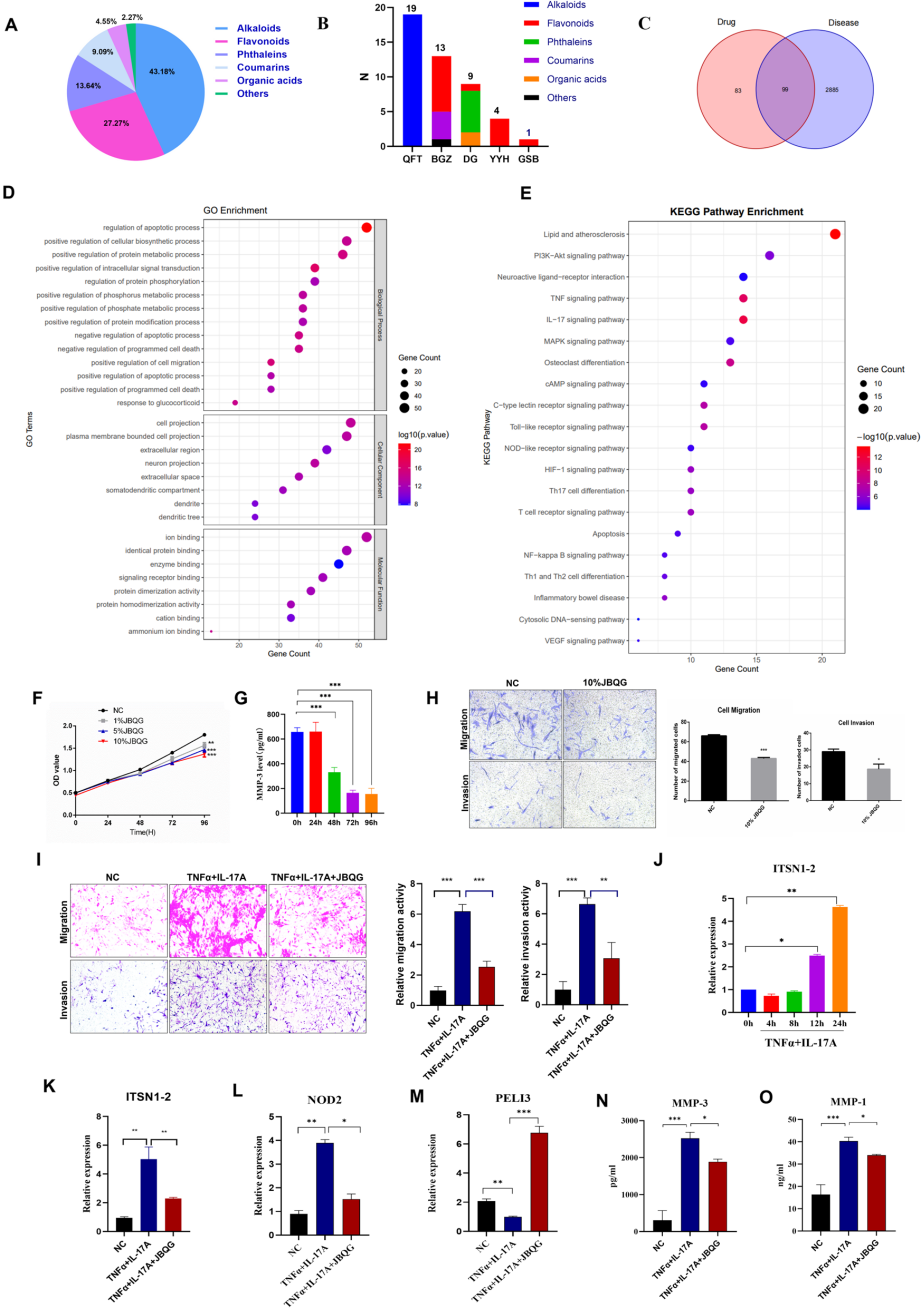
JBQG Regulates the lncRNA ITSN1-2/NOD2/PELI3 Signaling Axis. **A–C** Analysis of JBQG components absorbed into the bloodstream: A total of 44 active compounds were identified across six categories (**A**); Their distribution among the five constituent herbs of JBQG is shown (**B**); A Venn diagram demonstrates that 182 targets of these 44 compounds overlap with 2984 RA-related targets, identifying 99 shared targets (**C**). **D, E** Bioinformatics analysis of the 99 shared targets in RA: results include Gene Ontology (GO) enrichment analysis (**D**) and KEGG pathway analysis (**E**).** F–O** In vitro experiments validating JBQG’s effects on the lncRNA ITSN1-2/NOD2/PELI3 signaling axis: JBQG inhibited FLS cell activation in a time- and dose-dependent manner (**F**–**G**); JBQG significantly reduced the migration and invasion abilities of primary synovial fibroblasts (**H**) and MH7A cell lines (**I**); Stimulation of MH7A cells with 10 ng/mL TNFα and 10 ng/mL IL-17 for 0, 4, 8, 12, and 24 h caused a sustained increase in ITSN1-2 mRNA expression (**J**); After 24-h stimulation with TNFα and IL-17, MH7A cells exhibited significantly elevated ITSN1-2 (**K**) and NOD2 (**L**) mRNA levels, decreased PELI3 mRNA levels (M), and increased levels of MMP3 (**N**) and MMP1 (**O**) in the supernatant. These effects were notably alleviated by treatment with 10% JBQG-containing serum. Results represent three independent experiments. Comparisons between two groups were performed using T-tests, while one-way ANOVA with Tukey’s post hoc test was used for comparisons among three groups. *p < 0.05, **p < 0.01, ***p < 0.001, ***p < 0.0001 compared with the indicated group

Our previous studies have demonstrated that JBQG enhances clinical remission rates in RA patients and mitigates CIA-induced bone destruction in animal models [2, 3]. However, the molecular mechanisms underlying these effects-particularly their association with the NOD2/RIP2 signaling pathway-remain unclear. To address this, we combined mass spectrometry-based metabolomics and bioinformatics analysis to elucidate JBQG’s active components and potential therapeutic mechanisms in RA. JBQG’s blood-absorbed bioavailable components were identified via mass spectrometry, revealing 44compound primarily composed of alkaloids (43.18%, 19/44), flavonoids (27.27%, 12/44), and phthalides (13.63%, 6/44) (Fig. 2A, B). The alkaloids were exclusively derived from *Sinomenium acutum* (100%, 19/19), while flavonoids originated mainly from *Psoralea corylifolia* (66.67%, 8/12) and Epimedium brevicornu (33.33%, 4/12).

Using bioinformatics tools, we mapped these components to 182 target proteins, with 99 overlapping with RA-associated disease targets (Fig. 2C). Functional enrichment analysis highlighted pathways critical to RA pathogenesis: protein phosphorylation modification, phosphorus metabolism, cell migration, and apoptosis (Fig. 2D). KEGG pathway analysis further revealed significant enrichment in IL-17, TNF signaling pathway, osteoclast differentiation, Toll-like receptor signaling, and the NOD-like receptor signaling pathway, which are closely associated with inflammation and bone destruction (Fig. 2E). These data collectively suggest that JBQG’s potential to modulate the NOD2/RIP2 signaling pathway, a hypothesis supported by its overlap with known RA-associated targets.

To explore JBQG’s regulation of NOD2/RIP2 signaling, we investigated its effects on FLS. As shown in Fig. 2F, G, serum containing JBQG inhibited the proliferation and activation of primary synovial fibroblasts in a dose- and time-dependent manner. Treatment with 10% JBQG serum also suppressed migration and invasion of primary FLS and MH7A cell line (Fig. 2H, I), indicating anti-proliferative and anti-migratory effects. Previous studies established that lncRNA ITSN1-2 directly regulates NOD2/RIP2 signaling: ITSN1-2 knockdown reduces NOD2 expression, while NOD2 re-expression upregulates RIP2 and promotes FLS survival [7]. To link JBQG’s activity to this axis, we examined ITSN1-2 expression in JBQG-treated cells. Stimulation with pro-inflammatory cytokines(TNF-α and IL-17A) significantly elevated lncRNA ITSN1-2 expression (Fig. 2J), consistent with its role in inflammation. Conversely, JBQG treatment downregulated ITSN1-2 and NOD2 expression (Fig. 2K–L) while upregulating PELI3, an E3 ubiquitin ligase that promotes RIP2 ubiquitination and degradation (Fig. 2M). Additionally, JBQG reduced matrix metalloproteinases (MMP1/MMP3) levels (Fig. 2N–O), key mediators of joint destruction. These findings demonstrate that JBQG exerts its anti-arthritic effects through modulation of the lncRNA ITSN1-2/PELI3 signaling axis. By downregulating ITSN1-2 and NOD2 while upregulating PELI3, JBQG inhibits RIP2 activation, thereby suppressing NOD2/RIP2-mediated FLS proliferation, migration, and invasion. This mechanism not only alleviates synovial inflammation but also mitigates bone erosion, positioning JBQG as a promising therapeutic candidate for RA. Furthermore, the identification of PELI3 as a downstream target highlights its potential role in therapeutic strategies targeting ubiquitination-dependent pathways in inflammatory arthritis.

### lncRNA ITSN1-2 regulates NOD2/RIP2 signaling via a ceRNA network involving miR-6823-3p

**Fig. 3:**
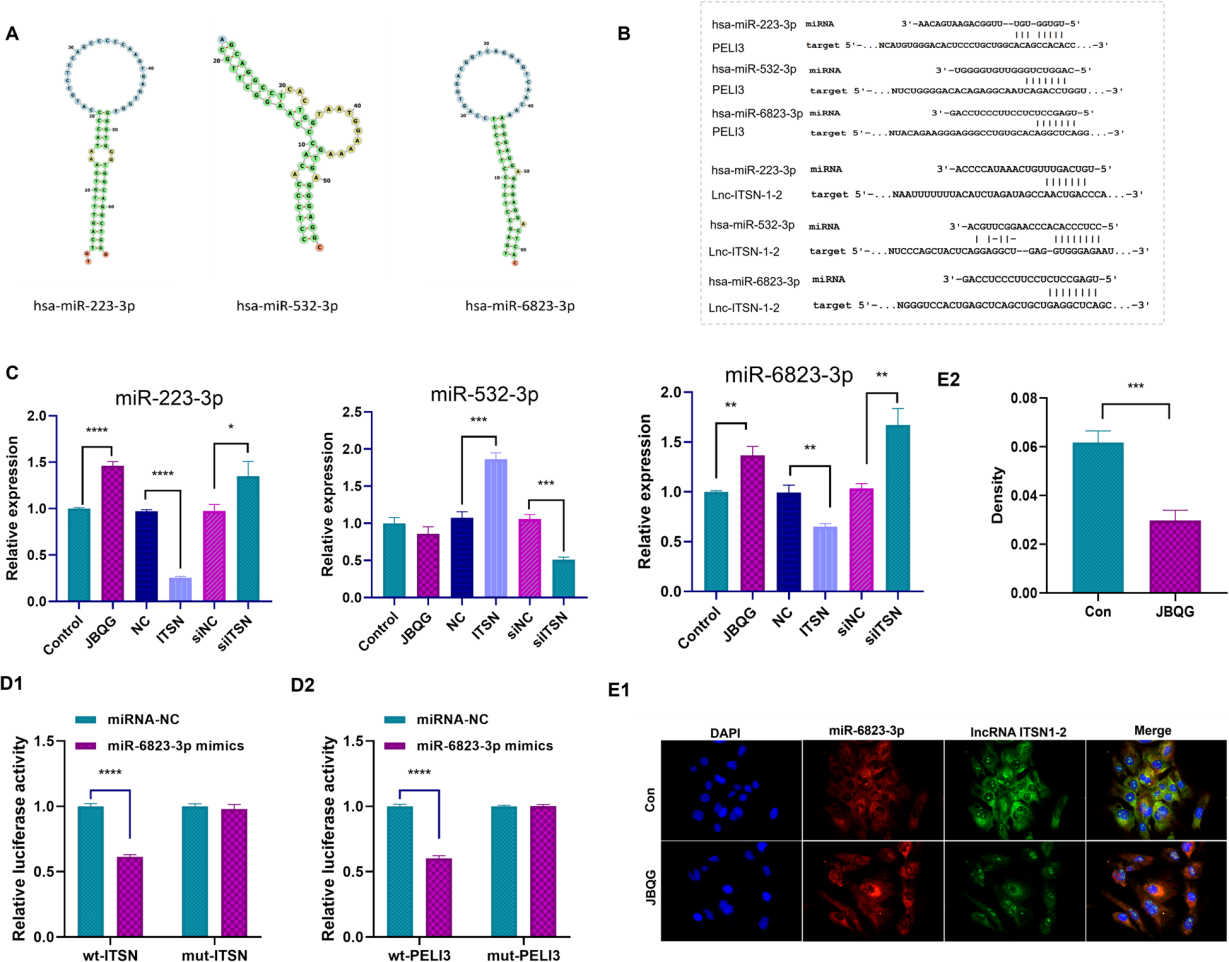
JBQG Suppresses lncRNA ITSN1-2 Expression and Disrupts Its Competitive Interaction with miR-6823-3p. **A, B** Bioinformatic analysis identified three miRNAs capable of binding both lncRNA ITSN1-2 and PELI3: miR-223-3p, miR-532-3p, and miR-6823-3p. **C** Regulatory effects of lncRNA ITSN1-2 and JBQG on miRNA expression in activated MH7A cells. MH7A cells were transfected with ITSN1-2 overexpression lentivirus (ITSN) or ITSN1-2 siRNA (siITSN) for 48 h, followed by stimulation with 100 ng/mL TNFα for 24 h and treatment with JBQG-containing serum (JBQG) or control serum (Control) for an additional 24 h. The expression levels of miR-223-3p, miR-532-3p, and miR-6823-3p were evaluated. **D1–D2** Dual-luciferase reporter assays confirmed the binding capability of miR-6823-3p to lncRNA ITSN1-2 (D1) and PELI3 (D2). **E1–E2** FISH assays visualized the localization of miR-6823-3p and lncRNA ITSN1-2 in FLS cells and demonstrated the regulatory effects of JBQG on their expression (magnification 400 ×). ITSN: ITSN1-2 overexpression lentivirus; siITSN: ITSN1-2 siRNA; wt-ITSN: wild-type ITSN1-2; mu-ITSN: mutant ITSN1-2; JBQG: 10% JBQG-containing serum; Control: 10% control serum. Results are representative of three independent experiments. Statistical analyses were performed using one-way ANOVA with Tukey’s post hoc test and two-way ANOVA with Tukey’s multiple comparisons. *p < 0.05, **p < 0.01, ***p < 0.001, ***p < 0.0001 compared with the indicated group

It is well established that lncRNAs can exert biological functions through various mechanisms, including acting as competitive endogenous RNA (ceRNA) to sponge microRNAs (miRNAs). Previous study identified PELI3 as a critical meditator in NOD2/RIP2 signaling pathway [14]. To dissect ITSN1-2’s regulatory mechanism, we performed bioinformatics analyses to identify miRNA that bind both ITSN1-2 and PELI3. Three candidate miRNAs emerged: hsa-miR-223-3p, hsa-miR-532-3p, and hsa-miR-6823-3p (Fig. 3A, B).

Using lentivirus constructs to overexpress or knockdown ITSN1-2, we observed reciprocal changes in miRNA levels: ITSN1-2 overexpression significantly downregulated miR-223-3p and miR-6823-3p expression while upregulating miR-532-3p (Fig. 3C). Conversely, ITSN1-2 knockdown reversed these effects, increasing miR-223-3p and miR-6823-3p levels while decreasing miR-532-3p. JBQG treatment mimicked ITSN1-2 knockdown, reducing miR-223-3p and miR-6823-3p but not affecting miR-532-3p (Fig. 3C). To confirm interactions, we performed dual-luciferase reporter assays. As shown in Fig. 3D, miR-6823-3p mimics significantly repressed luciferase activity in wild-type (wt) ITSN1-2 and PELI3 reporters but had no effect on mutated constructs (Fig. 3D), confirming its binding capacity to both RNAs. RNA fluorescence in situ hybridization (FISH) further revealed that: ITSN1-2 is predominantly cytoplasmic, with minimal nuclear localization and miR-6823-3p is exclusively cytoplasmic (Fig. 3E). JBQG suppressed ITSN1-2 expression in the cytoplasm but did not alter miR-6823-3p levels, suggesting its anti-arthritic effects stem from destabilizing ITSN1-2 rather than directly targeting the miRNA. This ceRNA network positions ITSN1-2 as a master regulator of NOD2/RIP2 signaling. By sponging miR-6823-3p, ITSN1-2 alleviates miR-6823-3p-mediated suppression of PELI3. JBQG mimics ITSN1-2 knockdown, downregulating ITSN1-2 and upregulating PELI3, suggesting its efficacy in suppressing pathogenic signaling.

### JBQG inhibits NOD2/RIP2 pathway activation via lncRNA ITSN1-2/miR-6823-3p/PELI3 axis by selectively promoting RIP2 K48-Ubiquitination

**Fig. 4:**
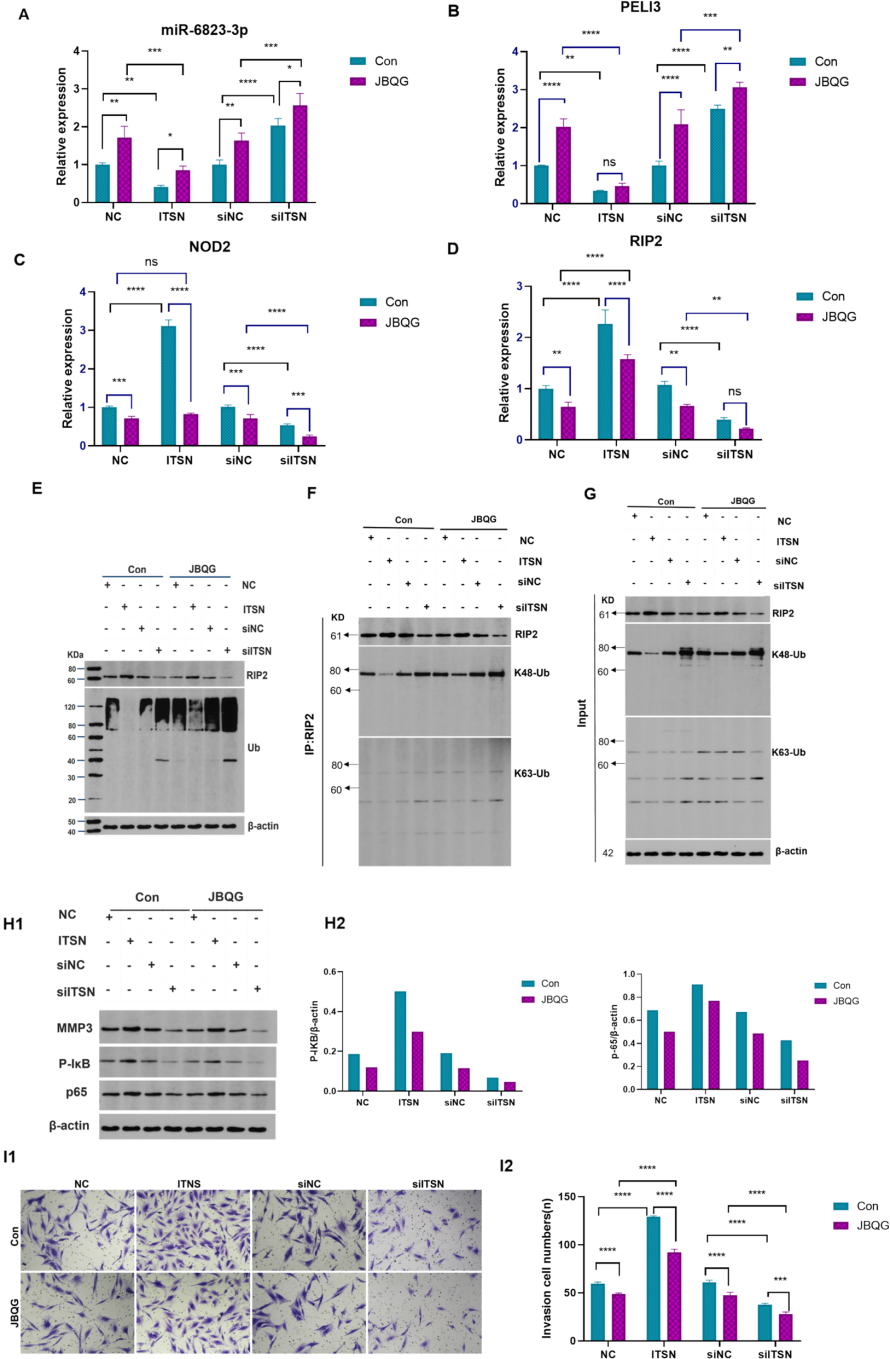
JBQG Suppresses lncRNA ITSN1-2 Expression, Enhances RIP2 K48-Linked Ubiquitination, and Inhibits NOD2/RIP2 Signal Activation. **A–D** Effects of lncRNA ITSN1-2 on miR-2683-3p, PELI3, NOD2, and RIP2 gene expression, and the regulatory role of JBQG. MH7A cell line were transfected with either ITSN1-2 overexpression lentivirus (ITSN) or ITSN1-2 siRNA (siITSN) for 48 h. Following transfection, cells were stimulated with 100 ng/mL TNFα for 24 h and treated with JBQG-containing serum (JBQG) or control serum (Control) for an additional 24 h. **E–G** Changes in RIP2 protein levels and K48/K63 ubiquitination. Total RIP2 protein and overall ubiquitination levels were analyzed via Western blot (**E**). Co-immunoprecipitation (CO-IP) assays evaluated RIP2 K48-linked and K63-linked ubiquitination (**F**, **G**). **H1–H2** Western blot analysis of MMP3, p-IKB, and IKB protein expression. **I1–I2** Effects of JBQG on cell invasion: JBQG significantly reduced the invasive capacity of MH7A cells in both the ITSN and siITSN groups. Statistical analyses were performed using two-way ANOVA with Tukey’s post hoc test for multiple comparisons. *p < 0.05, **p < 0.01, ***p < 0.001, ***p < 0.0001 compared with the indicated group

To further validate the role of JBQG in modulating the NOD2/RIP2 signaling pathway via lncRNA ITSN1-2, we performed lentivirus-regulated ITSN1-2 overexpress or knockdown in FLS cells followed by treatment with JBQG-containing serum or control serum. As shown in Fig. 4A–D, ITSN1-2 overexpression significantly downregulated miR-6823-3p and PELI3 expression while upregulating NOD2 and RIP2 expression. ITSN1-2 knockdown significantly upregulated miR-6823-3p and PELI3 expression while downregulating NOD2 and RIP2 expression. In the JBQG-treated group, miR-6823-3p and PELI3 expression were significantly upregulated, while NOD2 and RIP2 expression were significantly downregulated compared to the control serum group. However, the effects of JBQG were altered by ITSN1-2 overexpression or knockdown. In the ITSN1-2 overexpression cells, no significant change in PELI3 expression despite JBQG treatment, whereas in the knockdown cells, RIP2 expression remained unchanged post-JBQG. These data demonstrate that JBQG and ITSN1-2 exert opposing effects on the NOD2/RIP2 pathway, leading to functional compensation.

PELI3 is a critical E3 ubiquitin ligase responsible for inducing RIP2 ubiquitination. To investigate the effects of JBQG on RIP2 protein and its ubiquitinated forms, we analyzed RIP2 protein stability under ITSN1-2 modulation. As shown in Fig. 4E, ITSN1-2 overexpression significantly reduced RIP2 ubiquitination. Conversely, ITSN1-2 knockdown markedly increased RIP2 ubiquitination. JBQG treatment significantly elevated RIP2 ubiquitination in both ITSN1-2 overexpression and knockdown groups. Co-IP analysis further revealed that JBQG selectively enhanced RIP2 K48-linked ubiquitination, with no effect on K63-linked ubiquitination (Fig. 4F, G). These findings establish that JBQG promotes RIP2 degradation by K48-linked ubiquitination, dependent on ITSN1-2 suppression and PELI3 upregulation.

**Fig. 5:**
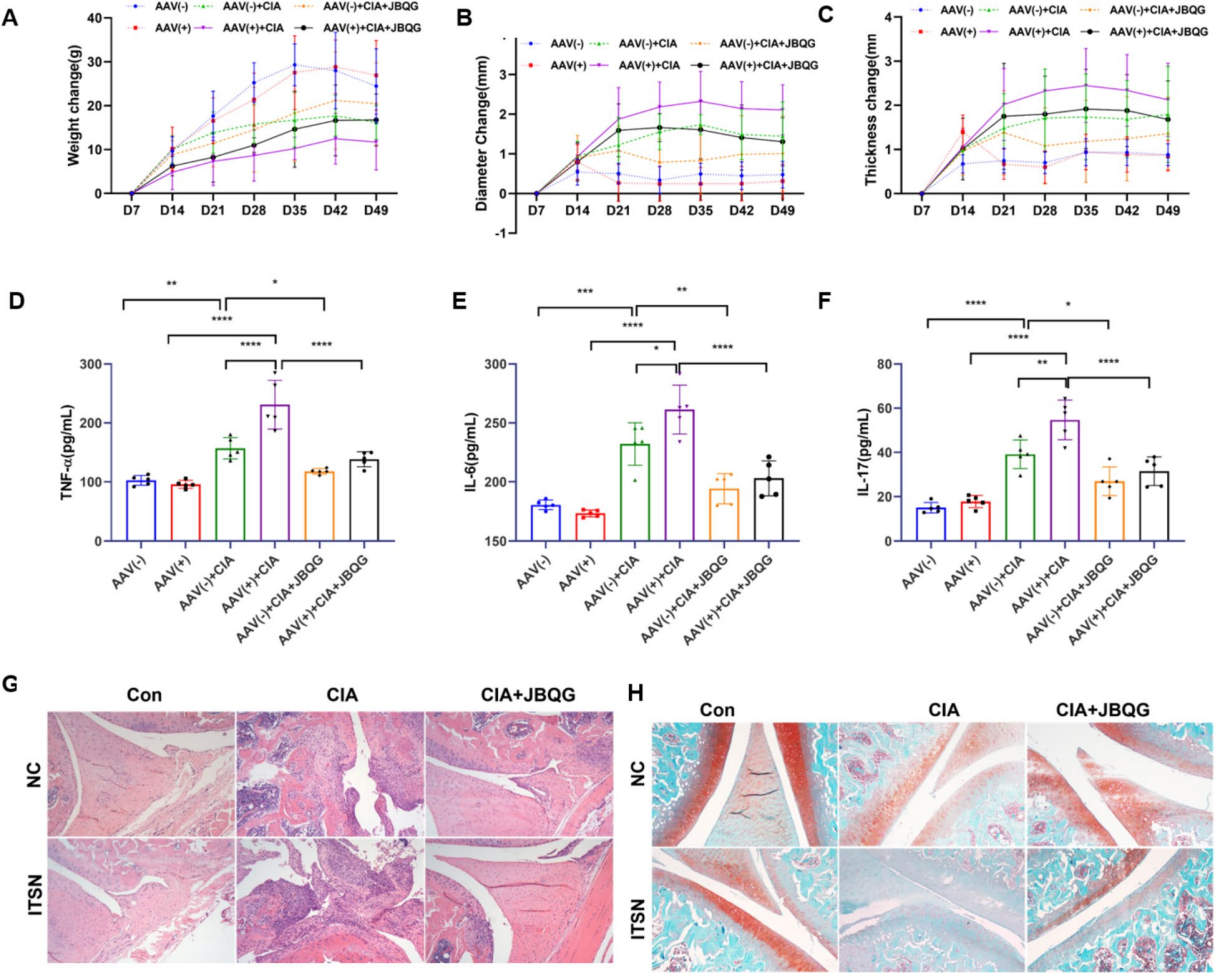
JBQG Significantly Alleviates Joint Inflammation and Cartilage Damage in ITSN Overexpression CIA Rats. **A–C** Changes in body weight, ankle joint diameter, and paw thickness among different groups of rats. **D–F** Peripheral blood levels of inflammatory cytokines (TNF-α, IL-6, and IL-17) in each group. **G** Hematoxylin and eosin (H&E) staining of knee joint tissues to assess inflammation and tissue damage (magnification × 100). **H** Safranin O/Fast Green staining of knee joint cartilage to evaluate cartilage integrity (magnification × 100). Statistical analyses were performed using one-way ANOVA with Tukey’s multiple comparisons.*p < 0.05, **p < 0.01, ***p < 0.001, ***p < 0.0001 compared with the indicated group

NOD2/RIP2 activation phosphorylates IκBα, triggering NF-κB nuclear translocation and pro-inflammatory cytokine release. To evaluate JBQG’s effects on these processes, we measured p-IKB, p65 and MMP3 protein levels, the latter being secreted by activated FLS cells. The results demonstrated that JBQG significantly downregulated p-IKB, p65 and MMP3 protein expression (Fig. 4H). In invasion assays, JBQG effectively inhibited FLS invasion in both ITSN1-2 overexpression and knockdown groups (Fig. 4I). These data confirm that JBQG’s anti-arthritic effects arise from RIP2 K48-ubiquitination-mediated suppression of NOD2/RIP2 signaling, reducing inflammation and MMP-dependent cartilage degradation. To translate findings to disease context, we analyzed ITSN1-2 overexpression in CIA rats. As shown in Fig. 5A–H, overexpression of ITSN1-2 in CIA rats significantly aggravated joint synovitis and increased cartilage destruction compared to CIA model rats, while JBQG treatment effectively reduced synovitis and cartilage damage. However, it is important to note that in the ITSN1-2 knockdown group in vitro (Fig. 4), JBQG treatment resulted in significantly reduced p-IKB levels, p65 and MMP3 protein expression, and FLS invasion compared to the control group. These results highlight JBQG’s therapeutic efficacy in RA, with potential benefits extending beyond ITSN1-2 modulation.

## Discussion

FLS cells are pivotal drivers of joint inflammation and bone destruction in RA. Their persistent proliferation and invasive migration directly contribute to synovitis and cartilage erosion, making them the "target organ" of RA progression. While immune cells such as T cells and macrophages orchestrate the inflammatory microenvironment, the pathogenic amplification of FLS-derived damage is uniquely linked to abnormal activation of NOD2/RIP2 signaling pathways and increased secretion of MMPs. In this study, we found that lncRNA ITSN1-2 as a critical regulator of FLS pathogenicity through its sponge effect on miR-6823-3p, leading to downregulation of PELI3 expression, reduced K48-linked ubiquitination of RIP2, activation of NF-κB signaling and downstream inflammatory cytokine release and enhanced MMP secretion and bone invasion. JBQG can inhibit the FLS hyperactivity and suppress bone-destructive cascades by downregulating lncRNA ITSN1-2 expression and restoring RIP2 K48-ubiquitination, which offers a dual mechanism for RA treatment.

Multiple studies have demonstrated that elevated expression of lncRNA ITSN1-2 in peripheral blood is significantly positively correlated with inflammatory responses and poor prognosis in various diseases, including sepsis [13, 15], acute ischemic stroke [12, 16], ankylosing spondylitis [17], and acute pancreatitis [18]. These findings suggest that lncRNA ITSN1-2 may serve as a universal biomarker for inflammation-driven pathologies. However, in RA patients, lncRNA ITSN1-2 expression exhibits striking heterogeneity between plasma and peripheral blood mononuclear cells (PBMCs). In peripheral blood, high lncRNA ITSN1-2 expression is positively correlated with disease activity indicators such as CRP, ESR, and DAS28 in RA patients, positioning it as a synovial inflammation specific biomarker. In contrast, in PBMCs, lower expression of lncRNA ITSN1-2 compared to healthy individuals and systemic lupus erythematosus (SLE) patients, with a negative correlation to CRP levels [19]. This discrepancy may result from the heterogeneity of PBMC populations, which comprise both pro-inflammatory and anti-inflammatory cell types, potentially masking clear trends [6]. It is known that lncRNA ITSN1-2 promotes the activation and proliferation of CD4^+^ T cells in peripheral blood, stimulates Th1/Th17 differentiation, and accelerates the inflammatory response [11]. However, its regulatory effects on anti-inflammatory and immunosuppressive cells such as Treg cells and γδ T cells remain unclear. Interestingly, it has been reported that RA patients using higher doses of glucocorticoids (> 7.5 mg/d) exhibit lower levels of lncRNA ITSN1-2 expression compared to those using lower doses (< 7.5 mg/d). But patients on DMARDs show higher lncRNA ITSN1-2 expression levels compared to those not using DMARDs [19]. High plasma ITSN1-2 correlates with RA activity, whereas low PBMC levels reflect immune subset heterogeneity. Glucocorticoids downregulate ITSN1-2, while DMARDs upregulate it, suggesting disease-stage-dependent effects.

Our research results reveal that the expression of lncRNA ITSN1-2 in the synovial tissue of RA patients is significantly elevated in RA synovial tissues compared to OA patients. Furthermore, its expression is positively correlated with RA disease activity indicators, including DAS28-ESR, CRP, and ESR, as well as with knee joint bone invasion scores. These findings are consistent with our previous results [6]. Additionally, our experiments confirm that inflammatory stimulation significantly further upregulates lncRNA ITSN1-2 expression in FLS cells, enhancing their migration and invasion capacities. These findings underscore ITSN1-2’s dual role as a pathogenic mediator and synovial-specific biomarker. The correlation between ITSN1-2 levels and bone invasion scores suggests its utility in monitoring RA progression and evaluating therapeutic efficacy, particularly in patients with active synovitis.

RIP2, a key adaptor protein in NOD-like receptor (NLR) signaling pathways, plays a central role in autoimmune inflammatory [20–23]. Small-molecule inhibitors targeting RIP2 have emerged as a new focus in drug development [23]. NOD2 recognizes the muramyl dipeptide (MDP) motif in bacterial peptidoglycans, activating and recruiting RIP2. The recruited RIP2 undergoes ubiquitination mediated by E3 ubiquitin ligases such as cIAP1, cIAP2, and XIAP. This process triggers downstream kinase signaling cascades, ultimately activating the MAPK and NF-κB pathways, which regulate a series of inflammatory responses [24].PELI3 is an important regulatory factor in the NOD2/RIP2 signaling pathway. The ubiquitin ligase encoded by PELI3 acts as a catalytic enzyme for RIP2 activation by directly binding to RIP2 protein and promoting its ubiquitination [14]. The ubiquitination of RIP2 at different sites can either activate or attenuate downstream NOD2/RIP2 signaling. Numerous studies have confirmed that increased K63-linked ubiquitination of RIP2 facilitates the recruitment of Transforming Growth Factor Beta-Activated Kinase (TAK)1and the IKK complex, thereby promoting NF-κB nuclear translocation and initiating the transcription of inflammation-related genes [25, 26]. Conversely, excessive K48-linked ubiquitination triggers a negative feedback mechanism that leads to RIP2 degradation, weakening its ability to activate NF-κB signaling [27, 28]. Our findings reveal that lncRNA ITSN1-2 sequesters miR-6823-3p via a sponge effect, targeting and suppressing PELI3 expression. Reduced PELI3 expression decreases K48-linked ubiquitination of RIP2, reducing its degradation, thereby activating the NOD2/RIP2 signaling pathway and triggering downstream inflammatory responses. NF-κB-driven cytokine release (TNF-α, IL-6) promotes FLS proliferation, migration, and MMP1/3 secretion, exacerbating bone erosion. These results suggest that lncRNA ITSN1-2’s sponge effect on miR-6823-3p represents a ceRNA-mediated negative feedback loop to amplify NOD2/RIP2 signaling. The data also demonstrate that miR-6823-3p positively regulates PELI3, diverging from its canonical gene silencing function [29].

JBQG, a traditional Chinese herbal compound, exhibits significant anti-arthritic effects. JBQG intervention significantly downregulates ITSN1-2 expression, restoring miR-6823–3-/PELI3 balance, and increases RIP2 K48-linked ubiquitination, promoting degradation and inhibiting NF-κB signaling. JBQG’s dual action on ITSN1-2 and RIP2 ubiquitination offers a mechanism-based therapy for RA, bypassing immune cell heterogeneity. Its efficacy in reducing MMP3 levels suggests potential to halt structural damage progression. Our findings indicates that the ITSN1-2/ceRNA axis may serve as a therapeutic target in NOD2/RIP2-driven diseases (e.g., cancer, RA, inflammatory bowel disease), but its inverse regulation in other disease like in SLE underscores the need for disease-specific biomarker panels.

However, our study has certain limitations. Does ITSN1-2 directly regulate bone remodeling? Preliminary data implicate FLS-derived MMPs, but its role in osteoblast function remains unclear. ITSN1-2 expression in distinct FLS subpopulations (e.g., pro-inflammatory M1 vs. anti-fibrotic M2) requires mapping via single-cell RNA-seq. In further experiments, in vitro bone metabolism models by co-culture FLS with osteoblasts/osteoclasts to dissect ITSN1-2’s direct effects on bone destruction are needed. More data are needed to assess JBQG’s efficacy in RA patients with high ITSN1-2 plasma levels in Phase II studies.

## Conclusion

**Fig. 6:**
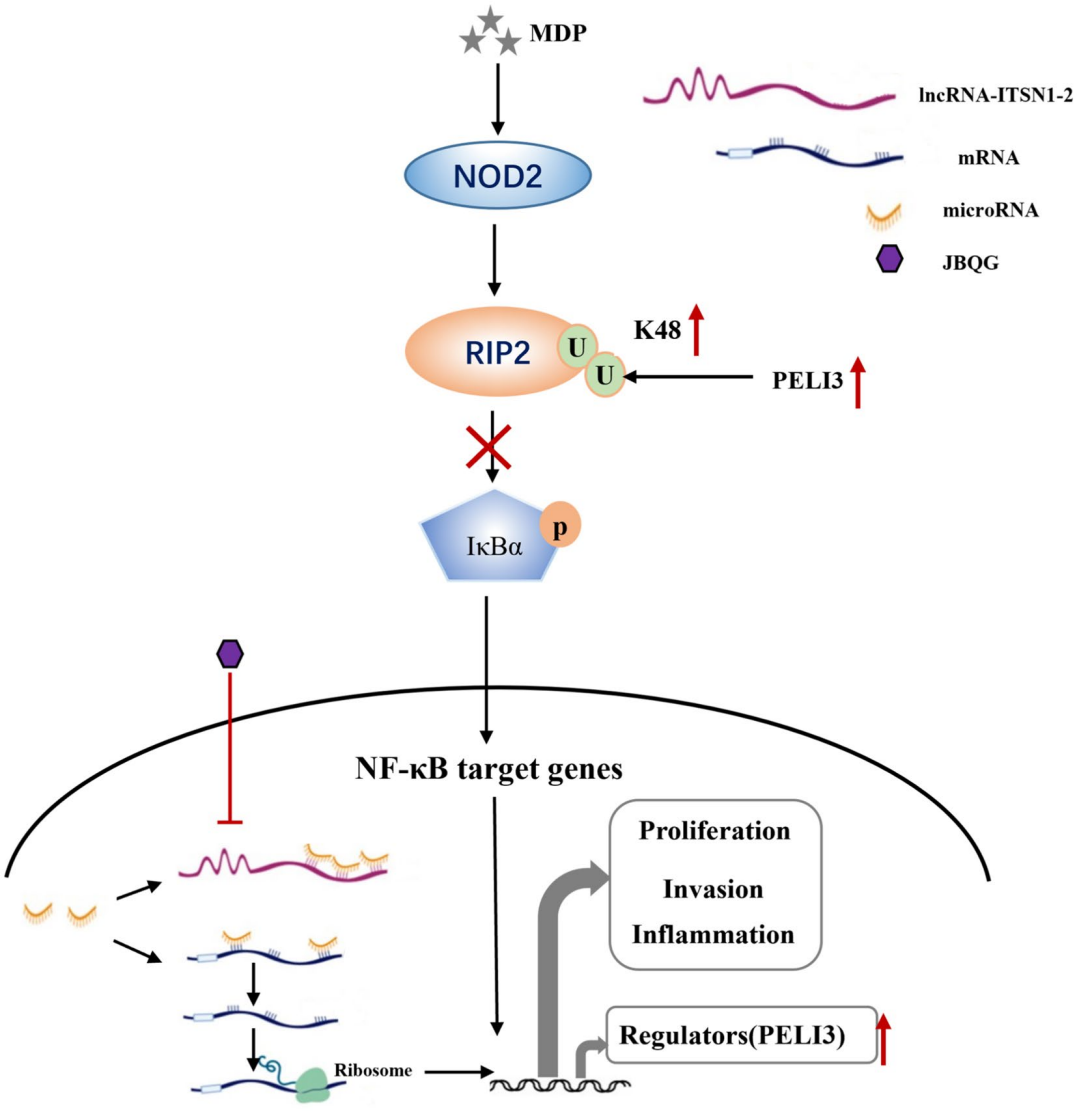
The JBQG regulates the lncRNA ITSN1-2/miR-6823-3p/PELI3 signaling axis in RA-FLS. Briefly, JBQG downregulates lncRNA ITSN1-2, which acts as a molecular sponge for miR-2863-3p, leading to the upregulation of PELI3 expression. This process induces an increase in RIP2-K48 ubiquitination, promoting its degradation and subsequently inhibiting the NOD2/RIP2 signaling pathway, thereby alleviating inflammation and invasive functions of RA-FLS cells. JBQG: Juanbi Qianggu Formula; RA: Rheumatoid arthritis; FLS: Fibroblast-like synovicytes

This study demonstrates that JBQG exerts potent anti-arthritic effects in RA therapy through dual regulatory mechanisms targeting the lncRNA ITSN1-2/miR-2683-3p/PELI3/RIP2 axis. JBQG reduces cytoplasmic lncRNA ITSN1-2 levels in FLS, derepressing miR-2683-3p to upregulate PELI3, which promotes RIP2 K48 ubiquitination and inhibits NF-κB signaling (Shown in Fig. 6). This dual action—suppressing upstream lncRNA-miRNA crosstalk and blocking downstream inflammatory cascades—not only alleviates synovitis and bone destruction but also enhances therapeutic efficacy by overcoming traditional single-target limitations. The findings highlight JBQG's potential as a precision medicine candidate for RA patients with high lncRNA ITSN1-2 expression and provide a mechanistic framework for combining TCM with biologics (e.g., NOD2 inhibitors) to achieve synergistic effects. Future studies should explore long-term safety profiling and clinical biomarker validation to advance RA treatment strategies.

## Supplementary Information

Additional file 1. **Fig. 1. Total ion chromatograms (TICs) of JBQG analyzed by UHPLC-Q-Orbitrap HRMS** (A) Negative ion mode; (B) Positive ion mode. **Supplementary Fig. 2. Total ion chromatograms (TICs) of control serum analyzed by UHPLC-Q-Orbitrap HRMS** (A) Negative ion mode; (B) Positive ion mode. **Supplementary Fig. 3. Total ion chromatograms (TICs) of JBQG-medicated serum analyzed by UHPLC-Q-Orbitrap HRMS** (A) Negative ion mode; (B) Positive ion mode.

## Data Availability

The datasets used and/or analyzed during this study are available from the corresponding author upon reasonable request.

## Abbreviations

CCK-8: Cell Counting Kit-8
ceRNA: Competitive endogenous RNA
CRP: C-reactive protein
DAS28-ESR: Disease Activity Score in 28 joints using Erythrocyte
ES: Bone erosion score
ESR: Erythrocyte Sedimentation Rate
FISH: Fluorescence in situ hybridization
JBQG: Juanbi Qianggu Formula
JSN: Joint space narrowing score
lncRNAs: Long noncoding RNAs
MDP: Muramyl dipeptide
MMP3: Matrix metalloproteinase 3
mSS: Modified Scoring System
NLR: NOD-like receptor
OD: Optical density
RA: Rheumatoid arthritis
TAK: Transforming Growth Factor Beta-Activated Kinase
TCM: Traditional Chinese Medicine
TLR: Toll-like receptor
