# Supplementary figures and images for: Juanbi Qianggu Formula inhibits fibroblast-like synovicytes activation via repressing LncRNA ITSN1-2 to promote RIP2 K48 ubiquitination
Source: Chin Med. 2025 Jul 8;20:109. doi: 10.1186/s13020-025-01164-4 (PMC12235879; doi:10.1186/s13020-025-01164-4)

**
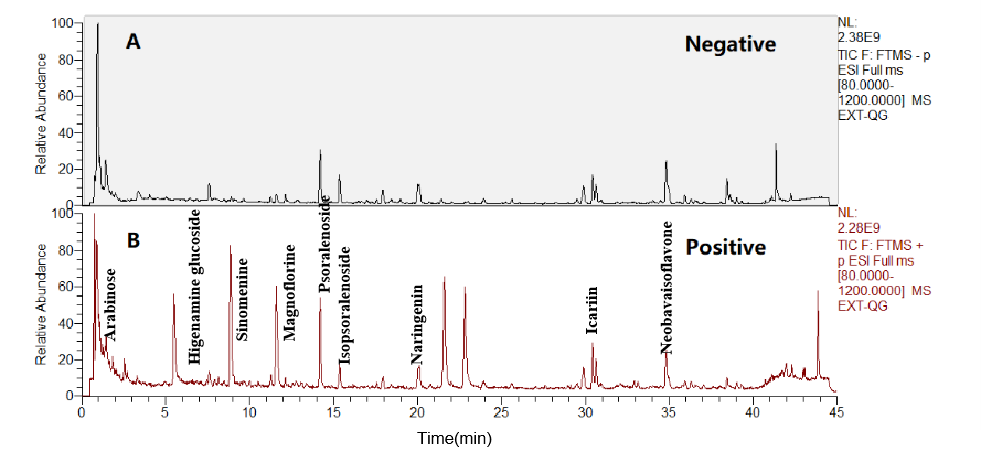
**

**Supple.Fig.1**

**
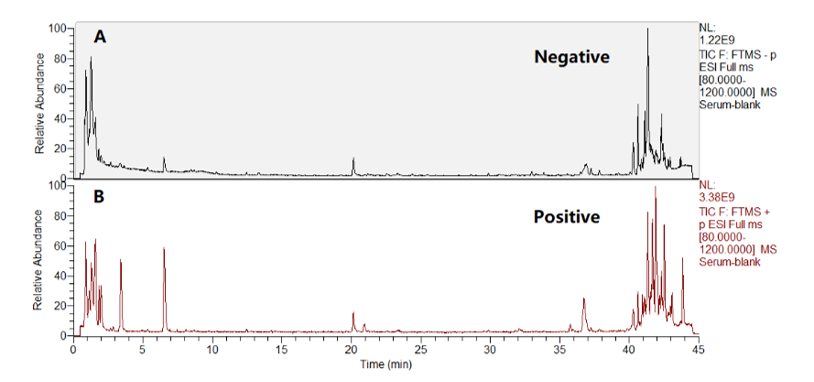
**

**Supple.Fig.2**

**
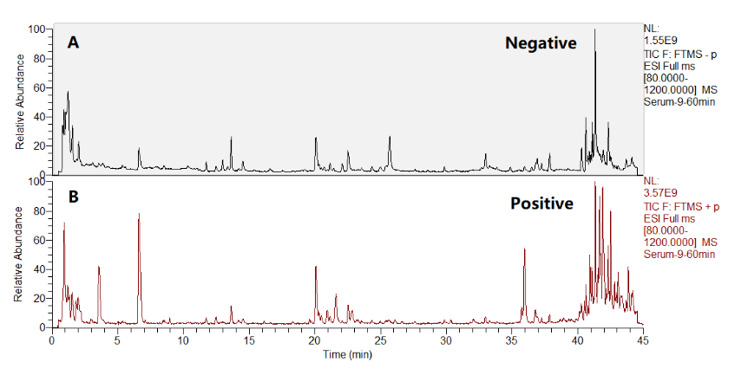
**

**Supple.Fig.3**

Supplement: Supplementary file 1 — Additional file 1. Fig. 1. Total ion chromatograms (TICs) of JBQG analyzed by UHPLC-Q-Orbitrap HRMS (A) Negative ion mode; (B) Positive ion mode. Supplementary Fig. 2. Total ion chromatograms (TICs) of control serum analyzed by UHPLC-Q-Orbitrap HRMS (A) Negative ion mode; (B) Positive ion mode. Supplementary Fig. 3. Total ion chromatograms (TICs) of JBQG-medicated serum analyzed by UHPLC-Q-Orbitrap HRMS (A) Negative ion mode; (B) Positive ion mode. [file 13020_2025_1164_MOESM1_ESM.docx]
